# Interactions between developmental phenology, carbon movement, and storage constrain demography in the understory clonal herb *Podophyllum peltatum* L.

**DOI:** 10.3389/fpls.2024.1325052

**Published:** 2024-06-26

**Authors:** Maxine A. Watson, Timo Vuorisalo

**Affiliations:** ^1^ Department of Biology, Indiana University, Bloomington, IN, United States; ^2^ Department of Biology, University of Turku, Turku, Finland

**Keywords:** developmental phenology, seasonal carbon integration, demography, development and demography, ^14^C-translocation, storage and integration, phenology

## Abstract

Little is known about how carbon integration and storage dynamics affect and are affected by demography in field populations. We sought to elucidate this link by examining dynamic patterns of carbon integration relative to the timing of demographically significant developmental decisions regarding shoot type determination in mayapple, *Podophyllum peltatum*, a clonal plant with large and persistent rhizomes. Individual rhizome systems growing in natural populations were fed ^14^CO2 either in late-April, early-May, or mid-June, then harvested at intervals throughout the current season and into the next. When distribution of label was examined we found that carbon fixed at different times in the growing season is used differently: April-fixed assimilate remained in the labeled shoot or was moved into the old rhizome, May-fixed assimilate was found predominantly in the old rhizome, while early-June fixed assimilate moved into the old rhizome and the extending new ramet. Movement of assimilate into the old rhizome appeared to have precedence over formation of additional new ramets. Despite significant within season changes in location of dominant sinks within rhizome systems, there was little redistribution of labeled assimilate: early fixed assimilate was not used to fuel later within season growth, however, assimilate was redistributed between seasons. Vegetative and sexual systems differed in the distribution only of April-fixed assimilate. This was observed even though early labeling occurred prior to anthesis. Sexual systems retained a greater proportion of assimilate in the stem than did vegetative ones, which exported more to the old rhizome. ^14^C-distribution patterns did not vary between systems differing in future demographic status suggesting that the developmental decision regarding shoot type is based on resources acquired in prior years. We explore the hypothesis that preformation and storage are functionally linked traits that permit plants to coordinate the developmental determination of structures differing in cost and demographic function with known resource status. We conclude that demography influences and is influenced by integrative physiology and that physiological restrictions on within season redistribution of assimilates constrain plants’ capacities to respond to short-term environmental variation. Such constraints may affect plants’ abilities to respond to rapid environmental change in the Anthropocene.

## 1 Introduction

The Anthropocene marks a period of man-made and rapid environmental change (Corlett, 2015). Limits on plants’ capacities to respond to such rapid change may be due either to constraints on plasticity and/or to the lack of appropriate genetic variation on which selection may act (e.g., Griffith and Watson, 2006; Dodd and Douhovnikoff, 2016; Kudoh et al., 2023; Williamson et al., 2023). Clonal plants may be particularly susceptible because clonality is at its heart a conservative growth strategy. It is of greatest advantage when environmental conditions are predictable. While observational data allow us to document changes in growth and demographic expression induced by environmental change, to move from documentation to prediction requires more nuanced understanding of how particular plants work. We need to know how plants’ developmental programs and their integrative physiologies interact to create a particular pattern of demographic expression (Diggle, 1994; Watson et al., 1995; Geber et al., 1997a, b; Meloche and Diggle, 2003; Goldberg et al., 2020) and how these interactions facilitate or constrain their capacities to persist in a rapidly changing world.

Plant growth is modular and earlier developmental events influence later ones through their effects on resource status and patterns of resource distribution (Watson, 1984; Diggle, 1994; Meloche and Diggle, 2003). In turn, temporal patterns of resource distribution influence subsequent developmental decisions (Diggle, 1994; Watson et al., 1995, 1997; Sachs and Novoplansky, 1997; Geber et al., 1997a; Worley and Harder, 1999; Lapointe, 2001; Weinig and Delph, 2001; Meloche and Diggle, 2003; Huber et al., 2004). Storage is another important component of resource status (Lubbe et al., 2021), yet little is known about how storage and storage dynamics affect and are affected by demography (Goldberg et al., 2020). To examine the interactions between resource dynamics, storage and demography requires information about temporal changes in demographic status, developmental phenology (i.e., the timing of meristem commitment to alternate demographic functions) and the integrative physiology of the plant; an approach we termed developmental ecology (Watson, 1984; Watson et al., 1997).

Work on crop species and a few native species indicates that pathways of resource flow within plants change with temporal changes in sink strength, type of sink and relative positions of organs – all of which result from developmental decisions regarding patterns of meristem determination made throughout the life of the plant (Marshall, 1990; Watson et al., 1995, 1997; Meloche and Diggle, 2003). Relatively little data of this kind exist for native species in their natural habitats and most address changes in sink strength due to shading or herbivory rather than developmental phenology (Flanagan and Moser, 1985; Jónsdóttir and Callaghan 1989; Tissue and Nobel, 1990a, b; Zimmerman and Whigham, 1992; Tissue et al., 1995; Babst et al., 2008; MaChado et al., 2013). However, phenological data about resource use and integration should provide insight into several key issues, including: (1) the differential use of resources gained at different times in the growing season; (2) the degree to which plants can reallocate resources gained at one time in the growing season to support later growth and sexual reproduction; (3) the effect of demographic status on these patterns; (4) the effects of variation in patterns of resource integration on developmental events that affect future growth form and demography and (5) plants capacities to respond to rapid changes in their environment.

Clonal plants provide convenient systems for examining these relationships. Not only are their behaviors governed by physiological and developmental rules that generally apply to all plants (Watson et al., 1995, 1997; Huber et al., 1999, 2004; Rashid et al., 2023) but they also maintain unique organs – stolons and rhizomes – that have important integrative functions (Jónsdóttir and Watson, 1997), provide storage capacity (Suzuki and Hutchings, 1997; Suzuki and Stuefer, 1999), and retain meristem banks that can be used in damage recovery (e.g., Eisen et al., 2021). The role of storage generally is evaluated indirectly or through examination of changes in total nonstructural carbohydrate (TNC) pools. Little is known, for example, about the developmental equivalency of carbon fixed and stored at different times in seasonal phenology or constraints on the remobilization of stored resources. Chapin et al. (1990) suggest that all stored carbon is not equally available at any given time in plants’ developmental phenology and data from other studies are consistent with this hypothesis (Jónsdóttir and Callaghan, 1989; Suzuki and Hutchings, 1997; Stuefer and Huber, 1999; Suzuki and Stuefer, 1999).

Approaches that take a dynamic perspective on patterns of resource use have proved valuable in other systems (e.g., Ashmun, 1994), and we take such an approach here. We address the link between the timing of demographically significant developmental events, current and future demographic status, and temporal patterns of resource movement by feeding the radioactive isotope ^14^CO_2_ to plants in natural field populations. Using a matrix of label and harvest dates, we test the following hypotheses: (1) Assimilate fixed at different times in the growing season is transported to different structures. (2) There is significant remobilization of assimilate fixed early in the season, when light levels are high, to support the growth of later-developing structures, when light levels are low. (3) Current and future demographic status of the aerial shoot influences seasonal patterns of assimilate movement within rhizome systems (physically and physiologically interconnected sets of ramets within a clone).

### 1.1 Study system

**Figure 1:**
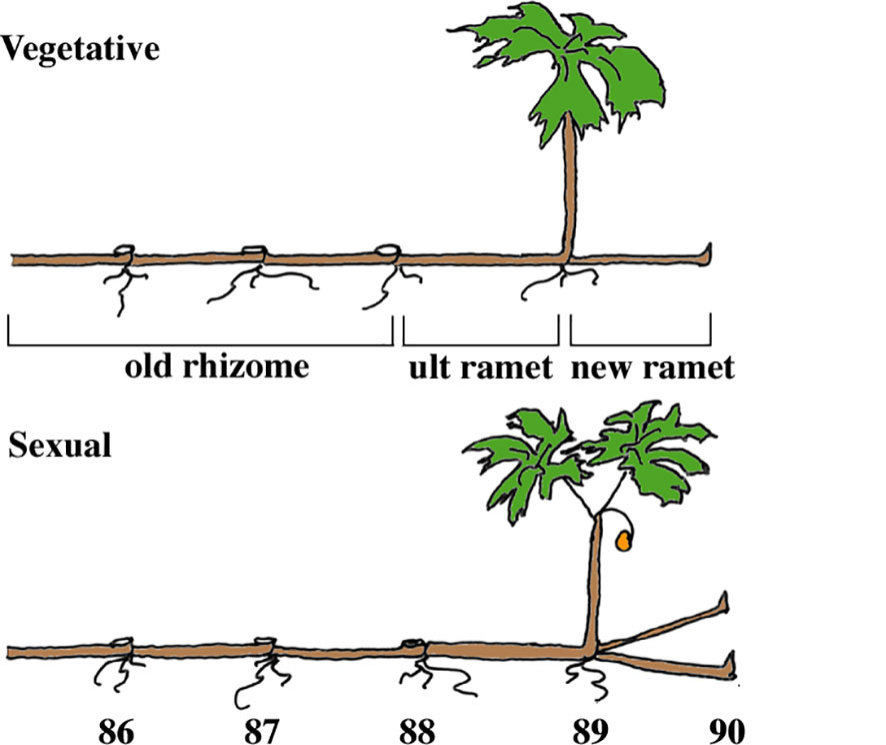
The architecture of vegetative and sexual rhizome systems of mayapple, *Podophyllum peltatum*. Plants are typical of those found in late June in Indiana. The four compartments used in the analyses are: new ramet, ultimate (ult) ramet, old rhizome, and shoot. In some analyses, the ultimate ramet is included in the old rhizome compartment. See text for explanation. The numbers beneath the ramets indicate the year in which each bore an aerial shoot.

We work with mayapple, *Podophyllum peltatum* L. (Berberidaceae), a long-lived rhizomatous clonal plant that is widely distributed in deciduous forests east of the Rockies in North America (Gleason and Cronquist, 1963). They grow in discrete patches of a few to many thousand aerial shoots belonging to one or more clones (Watson et al., unpublished). Mayapple rhizome systems consist of annually produced rhizome segments or ramets that remain morphologically and physiologically inter-connected generally for 6-7 years (Sohn and Policansky, 1977; Landa et al., 1992) (**Figure 1**). Most rhizome systems produce only one new ramet each year, rarely two or three (Geber et al., 1997a); but have the developmental capacity to produce up to five (Jones and Watson, 2001). In our experimental populations individual systems produced only a single terminal rhizome segment (ramet) per year. These newly forming ramets give rise to only one of two types of aerial structure; either a single vegetative leaf or a two-leaved sexual shoot that bears a terminal flower; the larger the terminal rhizome segment, the more likely it is to produce a sexual shoot, suggesting that the larger sexual shoot is more costly for the plant (Sohn and Policansky, 1977; Benner and Watson, 1989; de Kroon et al., 1991; Geber et al., 1997a, b).

Mayapples are obligately outcrossing but nectarless, and pollination rates of the most important pollinators, i.e., bumblebee queens (*Bombus* spp.) and honeybees (*Apis mellifera*) are usually low (Sohn and Policansky, 1977; Laverty and Plowright, 1988; Crants, 2008). The large fleshy fruits of mayapple are known to be dispersed by eastern box turtles (*Terrapene carolina*) (Rust and Roth, 1981). In our study area, another likely seed disperser is the raccoon (*Procyon lotor*). Raccoons feed on fruits of native vegetation in northern Indiana (Lehman, 1977) and are known to defecate germinable seeds (Willson, 1993; Niederhauser, 2015). Neither of these likely frugivores would aid in long distance dispersal. Insect herbivory in our study area was modest, but a few mayapple stems hosted stem boring larvae probably belonging to *Papaipema rutila* (cf. Bess, 2005).

Mayapple development is characterized by preformation, a common feature of plants of the deciduous forest understory (Foerste, 1891; Randall, 1952; Ott et al., 2019; Schnablova et al., 2020; Rünk et al., 2021; Rashid et al., 2023). New mayapple ramets are initiated almost two full years before their maturation aboveground (Watson et al., 1997; Geber et al., 1997a, b; Jones and Watson, 2001); they become morphologically determined as vegetative or sexual almost one full year before they emerge as aerial shoots and before the terminal bud reaches its final location on the forest floor and forms roots (Foerste, 1884; Geber et al., 1997a, b; Jones and Watson, 2001). The early determination of the demographic fate of aerial shoots almost one full year before their emergence aboveground suggests that the developmental decision to produce one or the other shoot type is contingent upon more than just the net amount of carbon accumulated by the developing terminal ramet within the season of shoot expansion. Further it suggests that assessments regarding the amount of assimilate fixed and stored in prior years also are important (Watson et al., 1997; Rünk et al., 2021; Rashid et al., 2023).

Because mayapple rhizome systems are highly integrated for carbon (Landa et al., 1992; Jónsdóttir and Watson, 1997) they, like other such plant species, should be able to move carbon stored in older ramets to developing axes, serving as a link between non-overlapping periods of high assimilate production and high carbon demand (Pitelka and Ashmun, 1985; Zimmerman and Whigham, 1992; Tissue et al., 1995; Wijesinghe and Whigham, 1997). In mayapple, integration for nitrogen-based resources is less extensive and more unidirectional with nitrogen-based resources generally moving from roots on intermediate-aged and new ramets to the developing terminal ramet (Jónsdóttir and Watson, 1997).

Mayapple rhizome systems exhibit extreme developmental division of labor among ramets, a phenomenon reported in an array of clonal plants (Alpert and Stuefer, 1997; Jónsdóttir et al., 1996). The current year’s ramet maintains both above and belowground function through the presence of aerial leaves and subterranean roots (**Figure 1**). All older ramets function only below ground and in the absence of injury to the rhizome system, these older ramets do not produce an aerial shoot again, but their roots do continue to function in mineral nutrient acquisition (Jónsdóttir and Watson, 1997) and the rhizome in storage. The older portions of rhizome systems also maintain a bank of dormant buds that is important in damage recovery (Watson et al., 1997; Ott et al., 2019; Eisen et al., 2021). Not surprisingly, in this understory herb carbon appears to be the critical limiting resource (Sohn and Policansky, 1977; Benner and Watson, 1989; de Kroon et al., 1991; Watson and Lu, 1999, 2004). We focus on carbon here.

These combined traits make mayapple a good subject for the study of the interaction between physiology, developmental phenology, and demographic determination. Mayapples grow slowly on relatively long-lived and highly integrated rhizome systems, and developmental determination of aerial shoot type takes place over an extended period of almost two years. This permits us to see interactions that would be difficult to detect but, no doubt, occur in faster growing taxa.

## 2 Materials and methods

Our study was conducted in an upland beech-maple-hickory temperate deciduous forest, on a private farm in Greene County, south-central Indiana (39°10’11”N, 86°42’58”W), the site of related work (Landa et al., 1992; Geber et al., 1997a; Watson and Lu, 1999, 2004). The study was initiated at the beginning of the 1989 growing season in several large colonies on a south-facing slope. Upon harvest we verified that there was little branching in our experimental rhizome systems, and most systems were made up of six to seven ramets, making these integrated subunits fairly uniform in age.

### 2.1 ^14^C-labeling and harvest protocols

Rhizome systems were labeled with ^14^CO_2_ either in late April, early May or early June and harvested either in mid-May, mid-June or September of 1989, or in mid-March of 1990, creating a matrix of seven label by harvest combinations that differed in the length of the chase period (**Table 1**). In late April (first label), mayapple shoots were almost fully expanded and the forest canopy was open. Two weeks later (May label/harvest), the forest canopy was closing and mayapple flowers were in anthesis. By June the forest canopy had been closed for several weeks and in this study all flowers and young fruits aborted.

**Table 1:** Matrix of label dates and length of the chase period, in weeks, used to examine the dynamic pattern of carbon movement in mayapple, *Podophyllum peltatum* L.

Harvest Dates					
	FL	FR	PS	ES	Total #
	May^a^	June	August	March	RhizomeSystems

Labeling Dates					
April (LE^a^)	2^b^	7	16		60
May (FL^a^)		5	14		40
June (FR^a^)			10	50	40

Total # Rhizome Systems					
Systems Harvested	20	40	60	20	140

^a^Labeling and harvest dates corresponded to the following periods in mayapple seasonal phenology: LE, leaf expansion; FL, flower anthesis; FR, fruit development; PS, post senescence; ES, early spring. Labeling dates were as follows: LE, April 25-28, 1989; Fl, May 5-8, 1989; FR, June 6-8, 1989. Harvest dates were as follows: FL, May 8-10; FR, June 8-15; PS, August 28-September 5; ES, March 22, 1990.

^b^Length of the chase period = number of weeks between labeling and harvest.

June is an important period in mayapple development. The new rhizome segment that will give rise to next year’s ramet experiences rapid extension growth (Geber et al., 1997a), the critical stages of morphological determination of the demographic status of next year’s aerial shoot occur (Jones and Watson, 2001), endogenous leaf senescence was first observed (Watson and Lu, 1999, 2004) and fruits when present enlarge. In late summer, the determination of next year’s shoot type, senescence of the current year’s shoot and expansion of the new rhizome segment are complete and the new ramet is forming roots. By March of the following year new aerial shoots are about to emerge above ground, with emergence regularly occurring in the first week in April at our location (Watson, pers. obs.).

For each label by harvest treatment combination, 10 rhizome systems bearing a vegetative and 10 bearing a sexual aerial shoot were labeled, for a total of 140 rhizome systems (**Table 1**). Selected shoots were sufficiently far apart that no two of them were interconnected within branching rhizome systems. This was confirmed when systems were harvested. Rhizome systems differed in the number of ramets making up the old rhizome, though virtually all systems had at least five old ramets. Label was introduced by exposing the single vegetative leaf, or the larger of the two leaves of sexual shoots to ^14^CO_2_ (100 µCi ^14^C per rhizome system (Amersham CFA.3, 50-60 mCi/mmole NaHCO_3_)) (Landa et al., 1992). Shoots that senesced prior to the scheduled harvest of their rhizome system were collected when they turned completely brown. At harvest, rhizome systems were washed, separated into individual ramets and their associated roots, and dried to constant weight at 65°C. For plants harvested in September 1989 and March 1990, the demographic status of the 1990 shoot (vegetative or sexual) was determined by dissection and recorded. The few plants that failed to incorporate ^14^CO_2_ were excluded from the translocation analyses but were used in the biomass analyses.

Following biomass determination, structures were ground in a Wiley mill using a 20 mesh, and two replicate subsamples of known weight oxidized in a Harvey Biological Oxidizer (model OX-400). Released ^14^CO_2_ was trapped in scintillation fluid (Harvey Biological ^14^C Cocktail) and the amount of label present in each subsample determined by scintillation counting (Landa et al., 1992). If the two subsamples gave similar values, they were averaged together, and that average value used to calculate ^14^C activity in each structure. If the two values differed, additional subsamples were run and included in calculations, but this was rarely necessary because subsamples showed a high degree of homogeneity. The resulting measure of activity per milligram dry weight specifies the specific activity. The total activity of a structure or compartment was calculated by multiplying its specific activity by its dry mass.

Labeled assimilate was used as a marker of the movement of total assimilate fixed at a given time. Data were aggregated into four compartments (**Figure 1**). (1) The ultimate old rhizome segment or ramet (Ult), which gives rise to the current year’s shoot. (2) The old rhizome compartment (Old), composed of all ramets proximal to (older than) the ultimate ramet. (3) The new ramet (New), is the ramet that morphologically differentiates next year’s aerial shoot during the current growing season, and (4) the current year’s aerial shoot (Shoot), which includes the leaves, petioles, stem and flower, if present. While the Ultimate, New and Shoot compartments were about the same age, the Old compartment, because it consisted of varying numbers of annual rhizome segments was not. Despite this variability in the size and age of the OLD compartment between rhizome systems, statistically significant differences were found. Root data were aggregated as described above. This differs from Landa et al. (1992) in which roots that were associated with the current year’s ramet were analyzed with those of the new ramet. Values of percent distribution were obtained by dividing the total amount of ^14^C in each compartment at the time of harvest by the total amount of label in the rhizome system at that time.

Specific activity was used as an indicator of differences among structures in sink strength. Because only the larger of the two leaves of sexual shoots was labeled, we normalized specific activity between vegetative and sexual rhizome systems by multiplying the specific activity of each structure in sexual systems by 1.67, the average ratio of total leaf area of sexual systems to the leaf area labeled. We assumed that seasonal photosynthetic profiles of sexual and vegetative systems were similar, which field gas exchange studies suggest is reasonable (Basha, Griffith, Carlson and Watson, unpubl.).

### 2.2 Data analysis

All analyses used the Fit Model module of JMP v 3.0 (SAS, 2000).

#### 2.2.1 Biomass

The labeling treatments did not affect overall rhizome system size or the distribution of biomass among compartments (analysis not shown) allowing data from the three labeling treatments to be combined within harvests and demographic categories for biomass analysis.

We performed two separate ANOVAs on the biomass of new rhizome and old rhizome compartments of the rhizome system (including the 1989 (Ult) ramet) to test for changes in biomass over time. We also performed planned comparisons between subsequent harvests and between vegetative and sexual systems within harvest. For statistical analysis, root and rhizome biomass was pooled within compartments as the analyses of the pooled data gave results similar to those for the un-pooled data with respect to harvest and shoot type effects.

#### 2.2.2 ^14^C distribution

The structure of the data sets on total ^14^C activity and specific activity are similar and, thus, the two analyses have many elements in common. MANOVA designs were used because multiple measurements of total and specific activity were made within rhizome systems. Repeated measures designs (Potvin et al., 1990) were used both because the within-rhizome ramet data is serially correlated due to the shared history of ramets within rhizome systems (Geber et al., 1997a, b) and ^14^C levels of ramets belonging to the same rhizome system are not independent because they are a function of the total amount of ^14^C taken up by each rhizome system. We used Pillai’s Trace because of its relative robustness and power (JMP v 4.0, SAS, 2000; Scheiner and Gurevitch, 2000); the more commonly used Wilke’s Lambda gave similar results.

Because not all combinations of label and harvest dates are represented in the experiment (**Table 1**), we performed five separate MANOVAs each for total and specific activity. Three of the five MANOVAs examined changes in labeled-assimilate distribution across the season for each label date separately, while the other two MANOVAS analyzed differences between label dates at two different harvests. However, because total activity and specific activity distribution were analyzed at different resolutions (total activity was examined across compartments, specific activity on a ramet-by-ramet basis), analyses of total activity and specific activity required different MANOVA designs.

*Total activity.* A sum design was used to examine between subject effects and a contrast design to examine within subject effects. Because all MANOVAs showed significant departures from sphericity, the degrees of freedom of all within subject tests were reduced using the Greenhouse-Geisser correction (JMP v 4.0, SAS, 2000; Scheiner and Gurevitch, 2000). Total activity data were grouped into the four compartments described above, with roots and rhizome pooled within each compartment. Overall differences among plants in the summed activity were reflected in the between-subjects effects: harvest dates (for MANOVAs by label date), label date (for MANOVAs by harvest date), and shoot type. All between-subject effects were treated as fixed factors. Differences in the distribution of activity within the plants were tested as within-subject effects. These included differences among compartments within individuals (compartment) and differences among individuals in the distribution of label among harvests, label dates or shoot types (Compartment * harvest, Compartment * label, Compartment * type effects respectively).

*Specific activity.* Analyses of specific activity differed from those of total activity in three ways: they involved only the below-ground organs, each ramet was analyzed separately, and the roots and rhizomes were analyzed separately, resulting in 14 dependent variables rather than just four as in the total activity analyses. These differences primarily affected the design of the within-subject portion of the MANOVAS, requiring a compound MANOVA design. There were two levels of belowground organ (root or rhizome) and seven levels of ramet (i.e., seven years of ramet growth) (**Figure 1**). Specific activities of missing organs, all of which were older ramets, were taken to be zero. We included root versus rhizome effects, ramet (i.e., positional) effects and their interaction as terms in the analysis. Between-subject effects were assessed with a sum design as described above for total activity.

## 3 Results

### 3.1 Biomass distribution

#### 3.1.1 Temporal changes in rhizome biomass as a function of current (1989) demographic status

**Figure 2:**
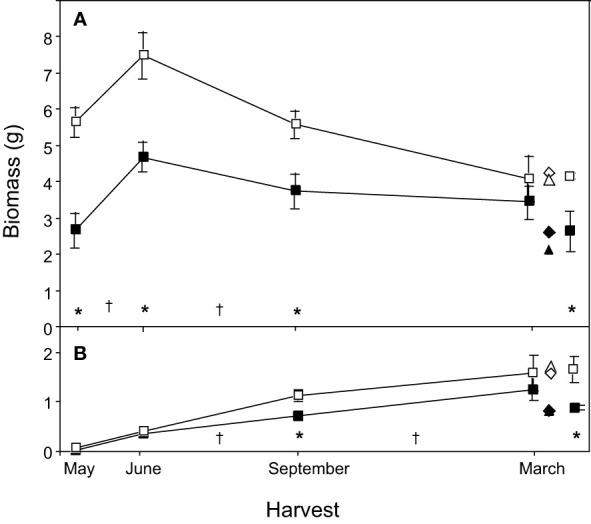
Changes in total rhizome biomass through time for vegetative and sexual rhizome systems of mayapple, *Podophyllum peltatum*. Biomass data for roots and rhizomes are combined, and data from the Ult ramet is included in the Old rhizome compartment. Data are represented as the mean ± 1 s.e. **(A)** Temporal changes in Old rhizome biomass for rhizome systems terminated in 1989 by either a sexual (□) or a vegetative (▪) shoot. Data from the March harvest is represented in three columns. The left-hand column represents data from rhizome systems that were terminated by either a sexual (□) or a vegetative (▪) shoot in 1989, as in earlier harvests. In contrast, the right-hand column represents data from rhizome systems that were terminated by either a sexual (□) or a vegetative (▪) shoot in 1990. The center column represents the life history status of each rhizome system in both 1989 and 1990: (open symbols represent rhizome systems that were sexual in 1990; closed symbols represent systems that were vegetative in 1990; (△) represent systems that were sexual in 1989; (♦), those that were vegetative in 1989. **(B)** Changes in biomass for the new ramet. Symbols as **(A)**. Asterisks designate statistically significant pairwise planned comparisons within harvests between systems differing in life history status (vegetative or sexual shoot in 1989); daggers (†) designate significant planned comparisons between harvest dates (see **Table 2**).

Old rhizome systems that were sexual in 1989 were heavier than those that were vegetative at each of the 1989 harvests (**Figure 2A**; **Table 2**, 1989 Type in May, June and September). This difference vanished by March 1990 when, instead, the demographic status of the 1990 shoot (1990 Type) drove the relationship (**Figure 2A**; **Table 3**).

**Table 2:** ANOVA of effects of Harvest Date and 1989 Life History Status (1989 Type, vegetative or sexual) on Old Rhizome Biomass (left) and New Rhizome Biomass (right)^1^.

Source	Old Rhizome Biomass				New Rhizome Biomass			
	DF	Sum Squares	F Ratio	Prob>F	DF	Sum Squares	F Ratio	Prob>F
1989 Type	1	178113361	23.3938	**< 0.0001**	1	1339823	4.1705	**0.0435**
Harvest	3	143115664	6.2657	**0.0006**	3	32128158	33.3355	**<0.0001**
1989 Type * Harvest	3	35238645	1.5428	0.2075	3	1228609	1.2748	0.2867

**Planned Comparisons**	**Estimate**	**t-Ratio**	**Prob. > |t|**		**Estimate**	**t-Ratio**	**Prob. > |t|**	
Harvest: May v June	-2700	-3.159	**0.0020**		-0.337	-1.952	(0.0535)	
Harvest: June v Sept	1671.9	2.7218	**0.0076**		-0.6888	-5.459	**<0.0001**	
Harvest: Sept v March	1504.5	1.9285	(0.0564)		-0.6351	-3.963	**0.0001**	
1989 Type: May	3983.4	2.8872	**0.0047**		0.0523	0.1844	0.8541	
1989 Type: June	3868.3	4.0248	**0.0001**		0.0531	0.2688	0.7886	
1989 Type: Sept.	2657.6	3.4726	**0.0007**		0.4890	3.1107	**0.0024**	
1989 Type: March	585.57	0.4306	0.6676		0.3679	1.3173	0.1905	

^1^See **Figure 1** – Vegetative for a description of the compartments.

Planned comparisons between subsequent harvests and between shoot types are shown in the bottom section of the table. Values in bold face are significant at p < 0.05; those in parentheses are of borderline significance.

**Table 3:** ANOVA of effects of 1990 Life History Status (1990 Type, vegetative or sexual) and Harvest Date (March v September) on Old Rhizome Biomass (left) and New Rhizome Biomass (right) ^1^.

Source	Old Rhizome Biomass				New Rhizome Biomass			
	DF	Sum Squares	F Ratio	Prob>F	DF	Sum Squares	F Ratio	Prob>F
Harvest (Mar v Sept)	1	44411160	6.7015	**0.0119**	1	2687927.2	6.2844	**0.0148**
1990 Type	1	142231739	21.4622	**<.0.0001**	1	9200633.3	21.5112	**<.0.0001**
Harvest (Mar v Sept) *1990 Type	1	78308	0.0118	0.9138	1	200039.9	0.4677	0.4966

**Planned Comparisons**	**Estimate**	**t - ratio**	**Prob > |t|**		**Estimate**	**t-ratio**	**Prob. > |t|**	
Harvest, Sexual: Sept v March	-2000	-2.179	**0.0331**		606.33	2.5776	**0.0123**	
Harvest,Vegetative: Sept v March	-1855	-1.579	0.1194		346.42	1.1605	0.2502	
1990 Type: September	3546.6	4.8669	**<0.0001**		751.39	4.0588	**<0.0001**	
1990 Type: March	3384	2.5901	**0.0119**		1011.3	3.0469	**0.0034**	

^1^See **Figure 1** – Vegetative for a description of the compartments.

Planned comparisons between subsequent harvests and between shoot types are shown in the bottom of the table. Values in bold face are significant at p < 0.05.

Old rhizome biomass increased by ca. 2.0g from May to June in both 1989 vegetative and sexual systems, but vegetative old rhizome biomass increased by 73% compared to 33% in the already larger sexual systems (**Figure 2A**; **Table 2**, 1989 Type: May contrast). The period of old rhizome biomass increase was followed by a period of progressive decline in biomass that extended over the remainder of the current growing season and into the next, for both vegetative and sexual rhizome systems. Systems that were sexual in 1989 showed a greater drop in average old rhizome biomass (3.4 g, 46%) than those that were vegetative (1.2 g, 26%), even though no fruiting occurred, resulting in a progressive convergence in old rhizome biomass between the two. By the following spring there was no difference in old rhizome biomass between systems that had been sexual versus vegetative in 1989 (**Figure 2A**; **Table 2**, 1989 Type: March contrast).

Increase in new ramet biomass was similar in 1989 vegetative and sexual systems (**Figure 2B**; **Table 2**, Shoot * Harvest). However, at every harvest, 1989 sexual systems produced new ramets that were larger than those produced by 1989 vegetative systems, although the differences were statistically significant only in September (**Figure 2B**; **Table 2**, 1989 Type: September contrast).

#### 3.1.2 Temporal changes in rhizome biomass as a function of future (1990) demographic status

When rhizome systems that were harvested in September 1989 or March 1990 were categorized by 1990 rather than 1989 shoot type different patterns of biomass distribution were found (**Figure 2A**; **Table 3**). Systems that gave rise in 1990 to sexual shoots had significantly heavier old rhizomes in both September and March (**Table 3**, Type contrast, **Figure 2A** for March harvest, right column), but the difference between the two was not as large as in 1989 (**Figure 2A**). Similarly, new ramets that would be sexual in 1990 were significantly larger than those that would be vegetative, and the difference was statistically significant at both harvests (1990 Type: September and March contrasts; **Figure 2B** for March harvest).

#### 3.1.3 Distribution of biomass among ramets within rhizome systems

**Figure 3:**
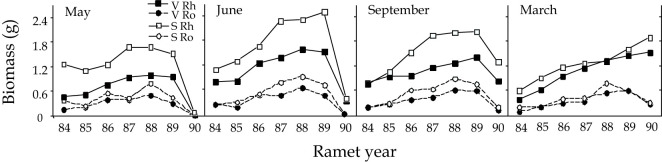
The distribution of root and rhizome biomass by ramet, at each harvest, for rhizome systems that were sexual or vegetative in 1989. The numbers on the X-axis represent the locations of ramets within rhizome systems, the number indicates the year that the ramet bore an aerial shoot.

Virtually all ramets of 1989 sexual systems were larger than vegetative systems at the May through September 1989 harvests, but they converged in size by March 1990 (**Figure 3**). Biomass shifted to the younger ramets between May and June and the shift was more striking in current (1989) sexual systems. From June through March, biomass was lost throughout the rhizome system, but particularly from the younger ramets of the old rhizome (**Figure 3**: 87, 88, 89 ramets). Although root and rhizome biomass were correlated (r = 0.4591), roots exhibited much less variation in biomass distribution among ramets from one harvest to the next. Considering our findings from the ^14^C data presented below, it is of particular interest that root biomass of the 1989 ramet did not increase significantly between June and September (F = 0.6435, p = 0.427).

### 3.2 Assimilate distribution

#### 3.2.1 The relationship between time of labeling and ^14^C distribution patterns

**Figure 4:**
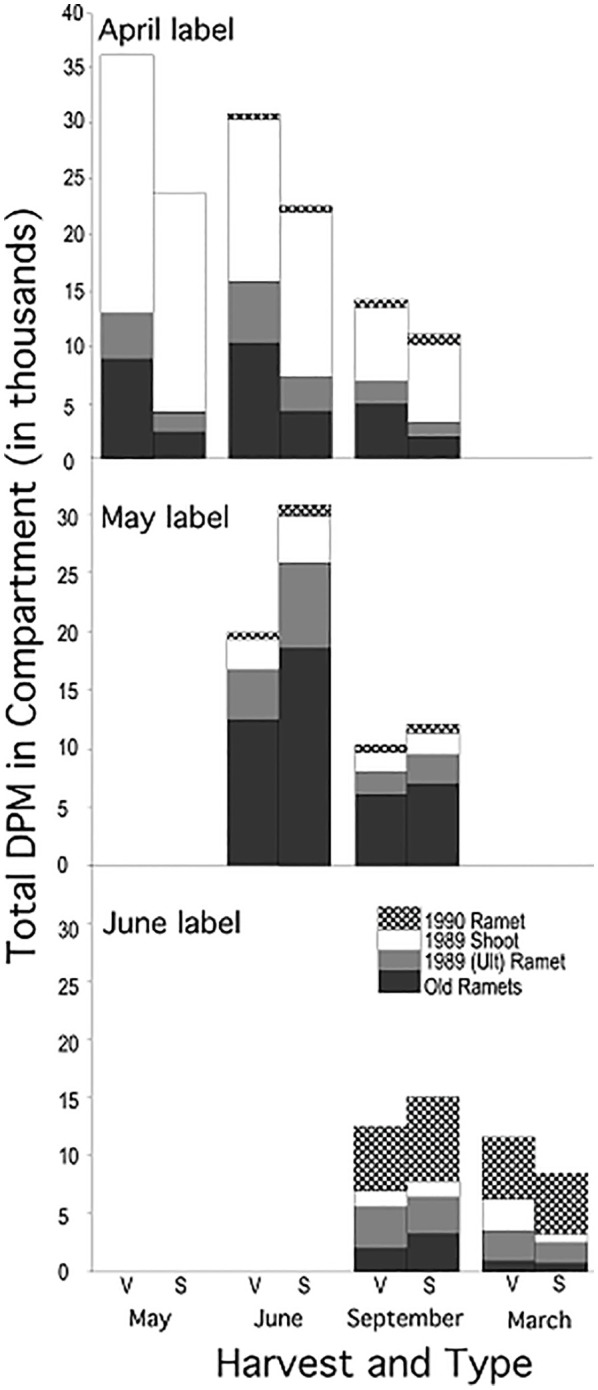
Distribution of mean total ^14^C activity among the four compartments of mayapple: New (1990) ramet, 1989 shoot, Ult ramet and old rhizome compartment (see **Figure 1**). Data for each label date are presented in separate horizontal panels: top (April), middle (May), bottom (June). Within each label date each harvest is represented as paired bars representing plants that were vegetative (left bar) or sexual (right bar) in 1989.

Plants labeled at different times in the growing season showed different patterns of label distribution (**Figure 4**, vertical comparisons, June and September harvests, **Table 4**, Compartment * Label Date): April label was found predominantly in the shoot (63% (V) to 80% (S)), May label in the old rhizome and ultimate rhizome segment (83%) and June label in the new ramet (45%) and old rhizome.

**Table 4:** Contrast MANOVA of the distribution of total activity by label date and 1989 Life history status (1989 Type, vegetative or sexual) for the June (left) and September (right) harvests.

	June Harvest				September Harvest			
Between Subjects [Sum]	DF	Pillai's Trace	Exact F	Prob>F	DF	Pillai's Trace	Exact F	Prob>F
Label Date	1, 29	0.0013	0.038	0.8471	2, 46	0.0161	0.377	0.688
1989 Type	1, 29	0.1452	4.926	**0.0344**	1, 46	0.1301	6.880	**0.0118**
Label Date * 1989 Type	1, 29	0.0681	2.119	0.1562	2, 46	0.1790	0.413	0.6636

**Within Subjects [Contrast]**	DF	**Pillai's Trace**	**Exact F**	**G-G P**	DF	**Pillai's Trace**	**Exact F**	**G-G P**
Compartment^1^	3, 27	0.7011	21.114	**<.0.0001**	3, 44	0.3660	8.464	**0.0011**
Compartment * Label Date	3, 27	0.5996	13.478	**0.001**	6, 90	0.9383	13.258	**<0.0001**
Compartment * 1989 Type	3, 27	0.1654	1.784	0.1928	3, 44	0.0845	1.354	0.2731
Compartment * Label Date * 1989 Type	3, 27	0.2925	3.720	(0.0643)	6, 86	0.2812	2.454	(0.0549)

^1^See **Figure 1** – Vegetative for a description of the compartments.

Between-subject effects reflect differences in total activity among rhizome systems. Within-subjects effects reflect differences in the distribution of label among the four compartments (New (1990 Root/Rhizome segment), Ult, 1989 ramet), old rhizome) within rhizome systems^1^. Values in bold face are significant at p < 0.05; those in parentheses are of borderline significance. Within subject tests have been corrected for departures from sphereicity using the Greenhouse-Geisser correction.

The demographic status of the current year’s shoot (1989, V or S) was a significant factor in how assimilate was distributed among compartments only for April-fixed assimilate; it was of marginal significance for May-fixed and non-significant for June-fixed assimilate. In systems labeled in April, sexual systems retained a greater proportion of label in their shoot (80% vs. 63% in vegetative systems) (**Figure 4**; **Table 5**, Compartment * 1989 Type). The effect of the 1989 shoot type diminished as the season progressed.

**Table 5:** Contrast MANOVAs of differences in the distribution of total activity by Harvest Date and 1989 Life history status (1989 type, vegetative or sexual).

	April Label				May Label				June Label			
Between-Subjects [Sum]	DF	Pillai's Trace	F	Prob>F	DF	Pillai's Trace	F	Prob>F	DF	Pillai's Trace	F	Prob>F
Harvest	2, 44	0.2495	7.314	**0.0018**	1, 30	0.2411	9.530	**0.0043**	1, 24	0.0403	1.010	0.325
1989 Type	1, 44	0.0232	1.043	0.312	1, 30	0.2410	9.530	**0.0043**	1, 24	0.1152	3.119	0.0901
Harvest * 1989 Type	2, 44	0.0009	0.021	0.9795	1, 30	0.0801	2.618	0.1161	1, 24	0.0113	0.275	0.605

**Within-Subjects [Contrast]**												
Compartment^1^	3, 42	0.768	46.356	**<0.0001**	3, 28	0.6466	17.080	**0.0020**	3, 22	0.4474	5.938	**0.0375**
Compartment * Harvest	6, 86	0.5357	5.244	**0.0102**	3, 28	0.3377	4.760	(0.0540)	3, 22	0.2838	2.907	0.1224
Compartment * 1989 Type	3, 42	0.3126	6.376	**0.0043**	3, 28	0.2986	3.974	(0.0742)	4, 19	0.2399	0.240	0.2666
Compartment * Harvest * 1989 Type	6, 86	0.0419	0.307	0.738	3, 28	0.1373	1.486	0.2507	4, 19	0.1914	0.191	0.3297

Analyses were performed separately for the April, May and June label dates. Each analysis is based on all available harvests of that label date. Between-subject effects reflect differences in total activity among rhizome systems. Within-subject effects test for differences in the distribution of activity among the four compartments within rhizome systems^1^. Values in bold face are significant at p < 0.05; those in parentheses are of borderline significance. Within subject tests have been corrected for departures from sphereicity using the Greenhouse-Geisser correction.

^1^See **Figure 1A** for a description of the compartments.

#### 3.2.2 Within and between season changes in patterns of assimilate distribution

There was little redistribution of label among compartments within the growing season irrespective of label date (**Figure 4**, horizontal comparisons). Statistically significant redistribution of label was noted for April-fixed assimilate, from the shoot into the old rhizome and new ramet compartments, and marginally significant redistribution for May-fixed assimilate (**Table 5**, Compartment * Harvest), but the magnitude of redistribution was small compared to the pronounced seasonal changes in dominant sink location (**Figure 4**, horizontal vs. vertical comparisons). The pattern of redistribution did not differ between 1989 sexual *vs* vegetative systems (**Table 5**, Compartment * Harvest * 1989 Type interaction).

While we saw little evidence of within season redistribution of label there was significant redistribution of label between the September 1989 and March 1990 harvests, from the 1989 old rhizome and ultimate rhizome segment into the 1990 ramet (**Figure 4**; **Table 5**).

Despite the small amount of within season remobilization of assimilate, rhizome systems did lose significant amounts of label through time: 10% between May and June, 50% between June and September, and 30% between September 1989 and March 1990 (**Figure 4** horizontal comparisons, **Table 5**, Harvest, April and May Label Dates), indicating that significant amounts of labeled carbon remain in metabolizable forms.

#### 3.2.3 Changing temporal patterns of specific activity among compartments

We examined changes in the specific activity of underground organs to detect whether ramets at different locations in the rhizome system, and their root and rhizome components, differed significantly in sink strength.

**Figure 5:**
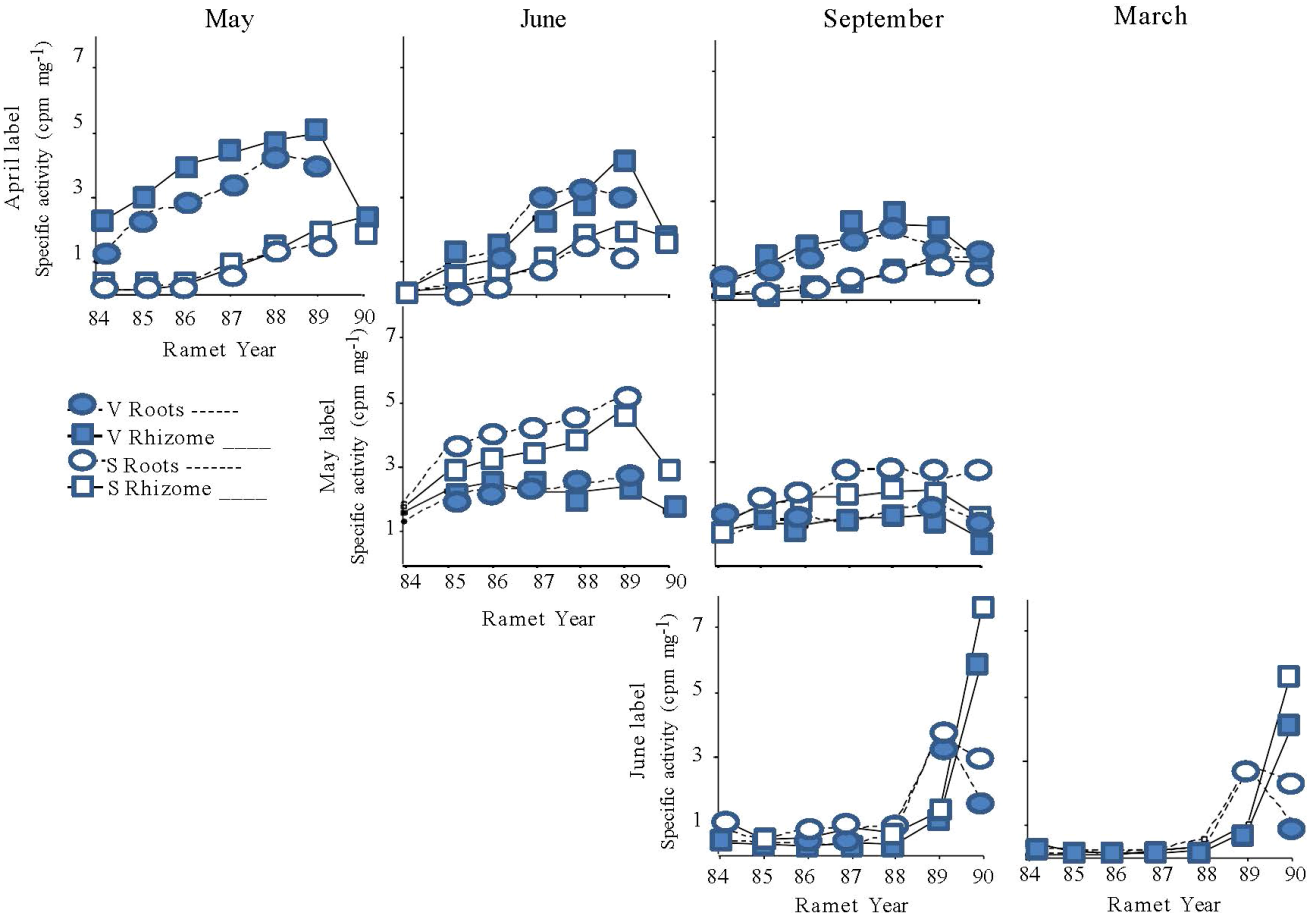
Specific activity of the below ground structures (roots = ⬭; rhizomes = □) of the ramets of mayapple in systems terminated by either a sexual (open symbols) or a vegetative shoot (closed symbols) in 1989. Rhizome systems were labeled at one of three times during the 1989 growing season: April (top panel), May (middle panel), or June (bottom panel), and then harvested either in May (far left), June (second from left) or September 1989 (second from right), or March 1990 (far right). The numbers on the X-axis identify the individual ramets within the rhizome system and correspond to the year in which each ramet bore an aerial shoot (**Figure 1**).

Seasonal changes in overall specific activity levels within rhizome systems mirrored the patterns observed for total activity. Rhizome systems labeled later in the season had lower overall specific activities than those labeled earlier (**Figure 5**; **Table 6**, Label Date) and overall specific activity levels generally decreased through time (**Table 6**, May and June Label, Harvest). The old rhizomes of 1989 vegetative systems were a stronger sink for April-fixed assimilate than those of 1989 sexual systems; while the reverse pattern was observed for May-fixed assimilate (**Figure 5**; **Table 6**, Label Date * Type; **Table 6**, Type).

**Table 6:** Compound MANOVA of specific activity distribution within rhizome systems in the June and September harvests as a function of Label Date and 1989 Life history status (1989 Type, vegetative or sexual).

Between Subject [Sum]	June Harvest				September Harvest			
	DF	Exact F		Prob>F	DF	Exact F		Prob>F
**Label Date**	1, 29	8.4647		**0.0069**	2, 46	2.592		(0.0858)
**1989 Type**	1, 29	0.1994		0.6585	1, 46	0.071		0.7908
Label Date * 1989 Type	1, 29	6.0451		**0.0202**	2, 46	2.783		(0.0723)

Within subject [Compound]	**DF**	**Pillai's trace**	**F**	**G-G P**	**DF**	**Pillai's trace**	**F**	**G-G P**
**Rt/Rhz**	1,29	0.146	4.968	**0.0337**	1,46	0.018	0.855	0.3600
Rt/Rhz * Label Date	1,29	0.053	1.614	0.2141	2,46	0.188	5.313	**0.0084**
Rt/Rhz * 1989 Type	1,29	0.009	0.269	0.6081	1,46	0.004	0.162	0.6895
Rt/Rhz * Label Date * 1989 Type	1,29	0.071	2.213	0.1476	2,46	0.016	0.369	0.6937
**Ramet**	6,24	0.738	11.29	**0.0027**	6,41	0.618	11.063	**0.0013**
Ramet * Label Date	6,24	0.575	5.422	**0.0254**	12,84	0.758	4.276	**0.0080**
Ramet * 1989 Type	6,24	0.124	0.568	0.5842	6,41	0.082	0.610	0.5571
Ramet * Label Date* 1989 Type	6,24	0.330	1.969	0.1902	12,84	0.284	1.159	0.3501
Rt/Rhz * Ramet	6,24	0.684	8.655	**0.0055**	6,41	0.340	3.514	(0.0876)
Rt/Rhz * Ramet * Label Date	6,24	0.317	1.860	0.2015	12,84	0.809	4.759	**0.0096**
Rt/Rhz * Ramet * 1989 Type	6,24	0.119	0.540	0.5975	6,41	0.087	0.651	0.4368
Rt/Rhz * Ramet * Label Date* 1989 Type	6,24	0.143	0.665	0.5336	12,84	0.329	1.378	0.2734

^1^See **Figure 1** for description of compartments. The between-subject analysis examines differences among rhizome systems based on a sum MANOVA design. Within-subject tests are a compound MANOVA with two levels of dependent variables (Root and Rhizome) for each of seven ramets.^1^ Within-subject effects test differences between roots and rhizomes (Rt/Rhz) within ramets, or differences between roots and rhizomes in the distribution among ramets (Rt/Rhz * Ramet). Values in bold face are significant at p < 0.05; those in parentheses are of borderline significance. Within subject tests have been corrected for departures from sphereicity using the Greenhouse-Geisser correction.

Significant differences in the specific activities of roots and rhizomes of individual ramets were detected in systems that were labeled in April or in May (rt/rhz: **Table 7**) but, for the most part, these differences were small (**Figure 5**) and were unaffected by 1989 shoot type (**Table 7**). However, for plants labeled in June, striking differences in the specific activity of roots and rhizome segments were found between the 1989 and 1990 ramets in both the September and March Harvests. The roots of the 1989 ramet labeled more heavily than the associated rhizome segment, while the opposite was true for the 1990 ramet (**Figure 5**, bottom panel).

**Table 7:** Compound MANOVA of specific activity distribution within the rhizome system for April, May and June label dates as a function of harvest date and 1989 Life history status (1989 Type, vegetative or sexual).

	April Label				May Label				June Label			
Between-Subject [Sum]	DF	Exact F	Prob>F		DF	Exact F	Prob>F		DF	Exact F	Prob>F	
**Harvest**	2, 44	2.612	0.0847		1, 30	6.221	**0.0184**		1, 30	5.982	**0.0205**	
**1989 Type**	1, 44	12.393	**0.0010**		1, 30	4.375	**0.0450**		1, 30	1.344	0.2555	
Harvest * 1989 Type	2, 44	1.687	0.1969		1, 30	1.021	0.3203		1, 30	0.309	0.5824	

Within Subject [Compound]	**DF**	**Pillai's tr.**	**F**	**G-G P**	**DF**	**Pillai's tr.**	**F**	**G-G P**	**DF**	**Pillai's tr.**	**F**	**G-G P**
**Rt/Rhz**	1,44	0.327	21.37	**<0.0001**	1,30	0.020	0.60	0.4439	1,30	0.201	7.54	**0.0101**
Rt/Rhz * Harvest	2,44	0.221	6.23	**0.0041**	1,30	0.131	4.51	**0.0421**	1,30	0.028	0.85	0.3642
Rt/Rhz * 1989 Type	1,44	0.044	2.01	0.1624	1,30	0.083	2.70	0.1107	1,30	0.003	0.09	0.7699
Rt/Rhz * Harvest * 1989 Type	2,44	0.098	2.40	0.1025	1,30	0.044	1.38	0.2487	1,30	0.002	0.06	0.8017
**Ramet**	6,39	0.699	15.05	**0.0003**	6,25	0.688	9.19	**0.0020**	6,25	0.729	11.20	**0.0155**
Ramet * Harvest	12,80	0.400	1.66	0.1838	6,25	0.486	3.93	**0.0363**	6,25	0.166	0.83	0.3976
Ramet * 1989 Type	6,39	0.371	3.83	**0.0452**	6,25	0.263	1.49	0.2676	6,25	0.212	1.12	0.3311
Ramet * Harvest * 1989 Type	12,80	0.373	1.53	0.2186	6,25	0.367	2.41	0.1175	6,25	0.163	0.81	0.4016
Rt/Rhz * Ramet	6,39	0.584	9.11	**0.0026**	6,25	0.439	3.27	0.0532	6,25	0.673	8.56	**0.0328**
Rt/Rhz * Ramet * Harvest	12,80	0.534	2.42	(0.0686)	6,25	0.620	6.80	**0.0047**	6,25	0.190	0.98	0.3689
Rt/Rhz * Ramet * 1989 Type	6,39	0.206	1.68	0.2192	6,25	0.161	0.80	0.5135	6,25	0.075	0.34	0.5860
Rt/Rhz * Ramet * Harv. * 1989 Type	12,80	0.197	0.72	0.5791	6,25	0.216	1.15	0.3635	6,25	0.153	0.75	0.4259

Between-Subject terms test overall differences among plants. See the caption of **Table 6** for explanation of abbreviations and terms. Values in bold face are significant at p < 0.05; those in parentheses are of borderline significance. Within subject tests have been corrected for departures from sphericity using the Greenhouse-Geisser correction.

#### 3.2.4 Relationship between patterns of assimilate movement and biomass changes within rhizome systems

The ^14^C data explain some aspects of biomass change better than others. The gain in biomass by the old rhizome compartment observed early in the season (**Figure 2A**) must result from the extensive movement of April- and May-fixed assimilate into the old rhizome (**Figure 4**). It is harder to explain the loss of biomass from the old rhizome later in the season (from June through September) and continuing at a reduced rate into the following year, in part because it is unclear what assimilate is being lost and how. Some of the carbon found in the old rhizome must be in a metabolizable form. Comparison of data from the short and long chase periods following the April and May labeling periods indicate that only a small, though statistically significant, amount of recently fixed assimilate could have been moved from the old rhizome into the new ramet over the course of the summer (**Figure 4**). We find that 79% of the May-fixed label remaining in the rhizome system in September is still found in the old rhizome (versus 84% in June), while only 6% of it is found in the new ramet (versus 3% in June), indicating that little remobilization from the old rhizome has occurred to build the new ramet. Further studies are needed before we can exclude the role of respiratory costs or mycorrhizae, as opposed to retranslocation, in the loss of old rhizome weight. These data suggest that the new ramet is constructed not only from current assimilate, as shown here, but by assimilate fixed in prior years. Longer-term studies are needed to address this question.

## 4 Discussion

By studying the dynamic patterns of carbon uptake and use within mayapple rhizome systems we have found apparent explanations for several demographic responses observed both by us in mayapple and in an array of other species. Our work demonstrates how the interaction between seasonal developmental phenology (i.e., the seasonal timing of meristem commitment), resource integration and storage both support and constrain plants’ capacities to respond to environmental variation. Further, we reveal constraints on the seasonal use of stored assimilate that can affect the timing and pathway of developmental decisions and, hence, demographic expression.

### 4.1 Storage and development

Mayapple behaves like a classic stress tolerator, foregoing growth for storage (Grime, 2001; Chapin et al., 1993). Up to 70% of recently fixed assimilate is allocated to the old rhizome rather than the production of additional new ramets, a developmental alternative for which meristems are available (Jones and Watson, 2001). Storage of large quantities of recently fixed assimilate is a common feature of perennial plants of seasonal environments (Chapin et al., 1990; Suzuki and Hutchings, 1997; Lapointe, 1998, 2001; Suzuki and Stuefer, 1999; Hart et al., 2024), with storage rates of 34-67% (Danckwerts and Gordon, 1987; Jónsdóttir and Callaghan, 1989; Wyka, 1999; Price et al., 2002; Sanz-Pérez et al., 2009). Yet, given the severely light-limited environment of the deciduous forest where mayapple grows, the amount of assimilate stored is striking. Our study finds that in mayapple, storage is an active process, in that it occurs simultaneously with growth and, hence, does not represent the passive accumulation of luxury resources (*sensu* Chapin et al., 1990).

Chapin et al. (1990) suggest that storage is selected under conditions where there is strong asynchrony between supply and demand, where risk of damage is high and/or when rapid shifts in the amount or nature of productivity occur, such as in the transition from vegetative to reproductive growth. Based on these assertions we predicted that in mayapple early fixed assimilate, which comprises the largest assimilate pool (Basha, Griffith, Carlson and Watson, unpubl.; this study, **Figure 4**), would be used to fuel late season growth, particularly the differentiation and extension of the terminal rhizome segment that forms the new ramet. However, we found that in mayapple there is little within season reallocation of recently fixed assimilate. Most of the assimilate that is moved into storage remains there at least until the end of the current growing season (**Figure 4**), but is remobilized to support growth of the new ramet in the following year (Landa et al., 1992); a pattern consistent with the findings of Eisen et al. (2021) on the response of mayapple rhizome systems to rhizome severing. Thus, our findings of temporal constraints on the use of stored assimilate more accurately support another thesis of Chapin et al. (1990) that stored carbohydrate is not necessarily available for use at any given time.

Such physiological constraints on within season remobilization of recently fixed assimilate appear to be widespread. Several studies in which leaf area, fruit set, or leaf shading were manipulated infer that recently fixed assimilate is unavailable to support short-term compensatory responses but is available in the long term (Zimmerman and Whigham, 1992; Cunningham, 1997; Wyka, 1999; Ehrlén and van Groenendael, 2001; Tixier et al., 2017; Eisen et al., 2021). In related work of ours Landa et al. (1992) also found significant reallocation of stored assimilate among years. This pattern appears to act as buffer that holds rates of fruit maturation constant when leaf area is manipulated (Sohn and Policansky, 1977). Further, we find that storage reserves are rapidly replenished by assimilate fixed in the current year (**Figures 4**, **5** and Landa et al., 1992), requiring bi-directional transport, a phenomenon observed in a variety of taxa from an array of habitats (Tissue and Nobel, 1990a, b; Jónsdóttir et al., 1996; Alpert and Stuefer, 1997; Jónsdóttir and Watson, 1997; Tixier et al., 2017).

Time lags of a year or more are commonly observed in responses of plants with extensive storage to short-term environmental variation (Zimmerman and Whigham, 1992; Cunningham, 1997; Geber et al., 1997a, b; Watson and Lu, 1999; Worley and Harder, 1999; Ehrlén and van Groenendael, 2001; Rünk et al., 2021). The failure to remobilize recently fixed assimilate within a growing season provides one explanation for the presence of such lags.

In mayapple, developmental phenology – when meristems are committed to alternate demographic functions - also appears to play a crucial role (Watson et al., 1995, 1997; Geber et al., 1997b) such that development phenology and integrative physiology work in concert to influence plants’ capacities to respond quickly to buffer environmental variation.

### 4.2 Preformation and storage

Plants that store significant amounts of assimilate also frequently exhibit preformation (Foerste, 1891; Randall, 1952; Lapointe, 2001; Schnablova et al., 2020; Rünk et al., 2021), a pattern of development in which organs are partially or completely determined one or more years before their expansion into mature structures (Watson et al., 1995, 1997; Diggle, 1997; Jones and Watson, 2001). The frequent co-occurrence of preformation with extensive storage suggests that the development and commitment of meristems to one or another structure is based upon the amount of assimilate stored, or being stored, at the time the developmental commitment is made. In this way, the plant could be assured of having sufficient resources to mature a particular structure, irrespective of subsequent environmental conditions. We see evidence of such a mechanism in mayapple, in which the new ramet is initiated two years (year X-2) before it emerges above ground (in year X) and one year before its rhizome elongates and the aerial shoot becomes irreversibly determined (in year X-1) (Watson et al., 1997). The elongation rate of the developing ramet in year X-1 is strongly correlated with the likelihood of that ramet differentiating a sexual shoot; ramets that elongate faster tend to be larger and are more likely to be sexual (Geber et al., 1997a). Interestingly, expanding ramets have a proclivity to grow faster or slower from the very onset of their growth in year X-1, suggesting that conditions, perhaps resource conditions at the time the bud is initiated (in year X-2), establish its growth trajectory in year X-1. Our data are consistent with the hypothesis that new ramet growth is supported by assimilate fixed in prior years (Eisen et al., 2021), given that assimilate fixed early in year X-1 (i.e., 1989) is not extensively used in the early stages of new ramet construction (Landa et al., 1992). Such a developmental program would match the pattern of meristem commitment with known levels of carbon resource availability, but at a cost, the loss of the capacity to respond quickly to changed environmental conditions (Watson et al., 1997; Worley and Harder, 1999; Werger and Huber, 2006). It is a pessimistic rather than an optimistic strategy (*sensu* Jones, 1992).

### 4.3 The role of current and future demographic status on carbon distribution and growth

Sexual and vegetative rhizome systems of mayapple differ in how they distribute assimilate, even in the absence of fruit initiation and maturation (**Figure 4**, April label). These differences could simply be caused by differences in the seasonal timing of developmental phenology, as reported between male and female plants of other species (e.g., Putwain and Harper, 1972; Watson, 1995; Laporte and Delph, 1996). But, in mayapple, differences in seasonal phenology between sexual and vegetative plants early in the season tend to be small (ca. 1-2 days) and are probably of insufficient magnitude to explain the differences between them in ^14^C-transport pattern. Phenological differences do increase later in the season - sexual shoots with fruit senesce on average one month later than vegetative ones (Watson and Lu, 1999, 2004), but because we lacked fruit-bearing sexuals in this study we could not assess how this difference affected carbon transport.

It also is possible that sexual shoots retain more assimilate in the leaf and stem in preparation for the possibility that a resource demanding fruit will be set; a form of short-term storage that has been observed by others (Chapin et al., 1990; Lapointe, 1998). If a sexual shoot fails to set fruit the unused assimilate could be re-allocated to other plant functions before the leaves senesce. In mayapple, sexual shoots that fail to mature fruit effectively become two-leafed vegetative shoots and they do give rise to disproportionately long new rhizomes (Sohn and Policansky, 1977), an observation consistent with this hypothesis. However, in mayapple, we do not see significant withdrawal of labeled assimilate from the senescing leaves of failed sexual shoots. Moreover, these failed sexual shoots senesce their leaves sooner than those that mature fruit (Watson and Lu, 1999, 2004), shortening their period of net positive photosynthesis and, resulting in the permanent loss of this locally stored assimilate when the leaf is shed, a potentially substantial and rarely considered cost to even unsuccessful reproduction. Despite loss of assimilate via senescence, mayapple sexual systems that fail to form fruit tend to be more robust in subsequent years than vegetative or successful sexual systems, perhaps owing to their greater leaf area alone. Not only do they produce larger new rhizome segments, they branch more frequently, and give rise to additional new sexual *vs* vegetative ramets (Sohn and Policansky, 1977; Geber et al., 1997a). Seemingly, the larger leaf area of the sexual shoot and its greater longevity (Watson, 1990; Watson and Lu, 1999, 2004) permits more assimilate to be fixed, thereby compensating for the higher loss of carbon by the senescing sexual leaf.

Interestingly, we found no significant differences in the patterns of distribution (**Figure 4**) and sink strength (**Figure 5**) of recently fixed assimilate between those systems that will become vegetative or sexual, even though larger new ramets tend to experience very different demographic fates (Sohn and Policansky, 1977; Geber et al., 1997a, b). These observations further support the idea that the performance of the new expanding ramet is based on the resources acquired in prior years and is consistent with the hypothesis that the preformation of structures influences the pathway of development based on the quantity of resources accumulated at the time of meristem determination rather than that of outgrowth.

### 4.4 Changing seasonal environments in the Anthropocene

Several issues remain unclear about controls on development and physiological integration in long-lived plants, of resource-poor environments, that may significantly affect their ability to rapidly respond to changing environmental cues in a warming world. In mayapple, for instance, we have a poor understanding of what triggers seasonal aerial growth. Observational data over many years indicates a one-week interval of emergence that is insensitive to temperatures experienced by plants during the preceding month (Watson, unpubl.), suggesting to us photoperiodic control. In contrast, the expansion rate of new rhizome segments *is* sensitive to environmental temperatures and thus can result in effects on flowering time (Watson, pers. obs.). Mayapple fertilization is dependent on outcrossing by insects, and rainy or cold conditions during flowering leads to reduced fruit set; typically less than 10% of sexual shoots mature fruit (Geber et al., 1997a). Thus, temperature and rainfall effects on phenology of either mayapple or its pollinators could have serious demographic consequences involving fruit set and, hence, the genetic diversity of local populations.

More closely aligned with the study reported here, we do not know what physiological mechanisms lead to movement of large amounts of assimilate into the old rhizome, nor do we understand what regulates the timing of this movement. Movement of resources into storage organs is generally thought to be controlled by sink-driven processes (Geiger, 1987) but, in mayapple, we have found that the largest concentration of newly fixed assimilate is in the oldest rhizome segment (Landa et al., 1992), (although this was less evident in the study reported here), and that segment is excised during the growing season and the assimilate it contains presumably lost. Arbuscular mycorrhizae also may play a role in creating sinks in mayapple rhizomes, but do not explain the oft-seen accumulation of label in the oldest rhizome segment, because mycorrhizae are found at highest concentrations at nodes closer to the younger end of the rhizome system (nodes X-4 to X-5) (Watson et al., 2001).

## 5 Concluding comments

Mayapple growth and development is characterized both by extensive storage and complete preformation of new structures. The co-occurrence of these two syndromes, one developmental and the other physiological, in stress tolerant organisms (Grime, 2001; Chapin et al., 1993) of strongly seasonal environments (Diggle, 1997; Watson et al., 1997) suggest that they act in concert to regulate the relative production of structures differing in demographic function and in long and short-term costs, based on estimates of resources on hand at the time the developmental commitment is made which, as in mayapple, is years earlier. These syndromes can be thought of as evolved solutions to problems posed by the physical and biological environments in which they are found (Watson et al., 1997; Watson, 2008). In mayapple, the interactions between developmental program and resource integration reflect a conservative growth strategy that is consistent with the constraints imposed by the environment in which they evolved; this strategy conserves resources over time but precludes rapid response to short term environment variation. Without knowing more about what factors govern the initiation of various developmental and physiological process – daylength, temperature, rainfall, symbionts – and how these may be affected by a warming habitat (e.g., changes in the timing of anthesis *vis-à-vis* the availability of pollinators; changes in the mycorrhizal symbiosis with changes in the intensities and patterns of rainfall), it remains difficult to assess the capacity of these tightly bound processes to continue to successfully interact and remain functional. For plants like mayapple, whose migration capacity is limited, the answers will be crucial to their persistence (Whigham, 2004).

## Data availability statement

The raw data supporting the conclusions of this article will be made available by the authors, without undue reservation.

## Author contributions

MW: Conceptualization, Funding acquisition, Methodology, Project administration, Writing – original draft, Writing – review & editing. TV: Investigation, Writing – review & editing.
